# A comparison of expressed emotion between mothers and their adolescent daughters with and without a history of nonsuicidal self-injury

**DOI:** 10.1186/s13034-022-00500-y

**Published:** 2022-08-08

**Authors:** Taru Tschan, Simone Pfeiffer, Raphael Gutzweiler, Tina In-Albon

**Affiliations:** Clinical Child and Adolescent Psychology, University of Koblenz-Landau, Landau, Ostbahnstraße 12, 76829 Landau, Germany

**Keywords:** Nonsuicidal self-injury, Expressed emotion, Five-minute speech sample, Adolescence, Family

## Abstract

**Background:**

Expressed Emotion refers to the extent to which close relatives express critical/hostile and/or emotionally overinvolved attitudes and feelings when speaking about a family member. High Expressed Emotion is a valuable predictor of clinical outcomes and is related to the presence of various mental disorders, including nonsuicidal self-injury (NSSI).

Interpersonal factors have been shown to be relevant in initiating and in maintaining with NSSI, as interpersonal difficulties are often reported as triggers for emotional dysregulation. The parental role in the etiology and treatment of NSSI needs to be further investigated. This study assesses Expressed Emotion in adolescents who engage in NSSI and their mothers.

**Method:**

We examined Expressed Emotion levels of mother–daughter dyads among adolescents who engage in NSSI, clinical controls (CCs), and nonclinical controls (NCs). The sample consisted of 70 female adolescents aged 12–20 years (*M* = 15.28 years, *SD* = 1.81; NSSI: *n* = 21, CC: *n* = 17, NC: *n* = 32) and 24 mothers aged 38–56 years (*M* = 46.47 years, *SD* = 4.61) using The Five-Minute Speech Sample (FMSS).

**Results:**

Adolescents who engage in NSSI exhibited significantly more covert criticism and critical tone toward their mothers than CCs (*d* = 0.65, *d* = 1.10) and NCs (*d* = 1.30, *d* = 1.10).

**Conclusion:**

The findings highlight the importance of family-based interventions for the treatment of NSSI in order to enhance a positive relationship quality between parents and adolescents.

## Introduction

Nonsuicidal self-injury (NSSI), the destruction of one’s own body tissue without intent to die, is highly prevalent among adolescents with lifetime prevalence rates between 18 and 39% [1, 2]. In a meta-analysis examining functions of NSSI, Taylor and colleagues [3] identified intrapersonal functions (66–81%), especially emotion regulation (63–78%), being the most frequent function, followed by interpersonal functions (33–56%). Interpersonal factors are relevant in initiating and maintaining NSSI, as interpersonal difficulties are often reported as triggers for emotional dysregulation [2]. In this context, NSSI can be understood as a dysfunctional emotion regulation strategy to deal with interpersonal stressors among other stressors associated with NSSI.

Nock’s [4, 5] comprehensive model of NSSI describes a range of distal risk factors (e.g., familial criticism/hostility, invalidation) and their impact on intra- and interpersonal vulnerabilities. According to this model, distal risk factors—such as an invalidating family environment—may lead to emotional dysregulation (intrapersonal consequence) as well as poor communication and problem-solving skills (interpersonal consequences) in individuals engaging in NSSI.

The development of emotion dysregulation—as an intrapersonal consequence of NSSI—through the interplay between an invalidating family environment and the child’s emotional reactivity has been described by the biosocial theory developed for Borderline Personality Disorder [6]. As NSSI is an emotion regulation strategy that is also a symptom of Borderline Personality Disorder, and also an adverse parent–child relational experience has been shown to predict greater emotion dysregulation, which in turn is associated with NSSI [7], and invalidating family environment might also apply to adolescents engaging in NSSI. Family interactions are reciprocally influenced by adolescents’ and parents’ behavior [8] and were found to be heavily shaped by conflict-ridden interaction patterns of adolescents who engage in NSSI and their mothers [9]. Intrapersonal and interpersonal consequences influence each other. Studies have suggested a mediating role of adolescent emotional regulation difficulties and poor coping strategies in the relationship between invalidating caregiving environments and NSSI [7, 10, 11], thus supporting Nock’s [4, 5] model of NSSI. Therefore, it seems to be of importance to examine the parent–child relationship in families with adolescents engaging in NSSI.

Expressed emotion is a measure of the family environment that describes the level of criticism, hostility, and emotional involvement that a relative expresses toward a family member [12, 13]. High expressed emotion predicts treatment discontinuation, relapse, and unfavorable disorder trajectories of various mental disorders in children, adolescents, and adults [14–17]. Maternal criticism appears to be associated with internalizing and externalizing disorders in adolescents, and emotional overinvolvement with internalizing problems [16]. However, there is also evidence that several of the scoring criteria for emotional overinvolvement have been adapted from parents and their adult children diagnosed with schizophrenia and might not be appropriate for parents of children with emotional and behavioral problems [18]. Maternal criticism, on the other hand, is a valid proxy for children’s psychopathology and an index of problematic parent–child interactions [19]. There is evidence that psychological disturbances contribute to increased expressed emotion and are also simultaneously maintained by expressed emotion [20]. High expressed emotion exhibited by parents is also related to the presence and frequency of suicidal ideation and plans, suicide attempts, and NSSI in youth [21]. On studying the relationship between expressed emotion and NSSI, parental criticism was associated with NSSI [21, 22], whereas emotional overinvolvement was not [21]. The association between parental criticism and NSSI was particularly strong among adolescents who were self-critical [21]. Further, maternal criticism was more strongly related to NSSI in girls than in boys [22]. Results relying on adolescents’ perceived levels of parental expressed emotion indicated that adolescents and young adults with a history of NSSI perceived their parents as less emotionally supportive, more intrusive, more irritating, and more critical than those without NSSI [23]. Similar results were found in adults engaging in NSSI [24]. In addition, adolescents’ self-criticism played a mediating role in the relationship between perceived parental expressed emotion and NSSI [23, 25]. Taken together, results indicate that the family environment of adolescents with NSSI seems to be characterized by high levels of criticism. Parental criticism may increase self-criticism in adolescents and thus the risk for NSSI [23]. However, the relationship between high expressed emotion (especially criticism) and NSSI in families of adolescents engaging in NSSI needs to be further explored. Previous research examining the link between expressed emotion and NSSI has focused on observed (coding of speech samples) and perceived (various self-report measures) levels of parental expressed emotion, neglecting the dyadic aspects of relationships. The conceptualization of expressed emotion as a unidirectional construct from parent to child may present an incomplete picture [26]. Further, a recent review on expressed emotion within families discusses its possible role in the transgenerational transmission of mental disorders [27].

### Aims of the study

So far, there is a lack of studies investigating expressed emotion in parent-adolescent dyads with the adolescent having a diagnosis of NSSI using the FMSS. Research to examine interpersonal difficulties as initiating and maintaining factors of NSSI is necessary to develop interventions to reduce NSSI. The parental role in the treatment of NSSI also needs to be investigated by studying the parent–child relationship. Expressed emotion is a highly relevant construct in families of adolescents with NSSI, however, studies examining the associations between expressed emotion levels of adolescents with NSSI and their parents are rare. Further, previous studies have focused on just one person’s perspective, either that of the parent [21, 22] or that of the adolescent [23–25]. The present study examines expressed emotion levels in female adolescents with NSSI and their mothers using the FMSS [28]. The primary aim of this exploratory study was to examine levels of expressed emotion in adolescents with NSSI toward their mothers as well as the level of expressed emotion in mothers toward their daughters with NSSI and compare the results with clinical and nonclinical mother-daughter dyad control-groups. We expected that adolescents of mothers with high expressed emotion would exhibit high expressed emotion. Furthermore, we were interested to learn if these mother-daughter dyads exhibited higher levels of expressed emotion than mother-daughter dyads from clinical and nonclinical control groups when administered the FMSS.

## Method

### Participants

#### Adolescents

The study included 70 female adolescents aged 12–20 years (M = 15.28 years, SD = 1.81). Twelve percent (n = 8) were aged between 18 and 20 years old. In terms of education, 72% attended a secondary school (58% attended a school for higher education), 14% attended a vocational school and 14% finished school with a high-school degree (“Abitur”). Twenty-one participants met the DSM-5 proposed research criteria for nonsuicidal self-injury behavior disorder diagnosis, 17 adolescents met the criteria for other mental disorders except the proposed NSSI disorder and were assigned to a clinical control group, and 32 adolescents did not fulfil the criteria of any mental disorder and were assigned to the non-clinical control group.

The most frequent comorbid mental disorders among adolescents with NSSI were depressive disorders (56.8%), anxiety disorders (17.7%), and posttraumatic stress disorder (11.8%). The CC group most frequently met the criteria for anxiety disorders (70.6%). CC adolescents (Mage = 16.12 years) and NC adolescents (*M*_age_ = 14.75 years) differed with respect to age (p < 0.05).

### Mothers

Twenty-four mothers aged 38–56 years (M = 46.47 years, SD = 4.61) participated, 10 in the NSSI group, 7 in the CC group, and 7 in the NC group.

#### Measures

Diagnostic Interview for Mental Disorders in Children and Adolescents. To examine the adolescents’ current or past DSM-5 diagnoses, clinical structured interviews were conducted by certified psychologists. The Diagnostic Interview for Mental Disorders in Children and Adolescents (Kinder-DIPS) [29] assesses the most common mental disorders in childhood and adolescence. The Kinder-DIPS has good validity and reliability (child version, κ = 0.48–0.88; [30]. The proposed NSSI disorder diagnosis (DSM-5, APA, 2013) was assessed with the additional NSSI section of the Kinder-DIPS [29]. Interrater reliability estimates for the diagnosis of NSSI were very good (κ = 1.00).

### FMSS

The FMSS [28] was used to assess expressed emotion levels and was administered individually wherein the adolescent and mother were interviewed separately. Adolescents and mothers were asked to speak for five uninterrupted minutes about their mother and daughter respectively and how they get along with each other. Participants got the following instructions:

I’d like to hear your thoughts about your (mother/daughter) in your own words and without my interrupting you with any questions or comments. When you begin, I’d like you to speak for 5 min, telling me what kind of person your (mother/daughter) is, and how the two of you get along together. Once you have started, I will not be able to answer any questions. Is there anything you would like to ask before you begin? The monologues were videotaped and coded for relationship quality, criticism, covert criticism, and emotional overinvolvement [31, 32] by two trained independent raters who were blinded to group allocation. Relationship quality was rated as positive, neutral, or negative.

Criticism refers to statements expressing unambiguous dislike, disapproval, or resentment of the relative’s behavior or personality and was coded based on content and/or tone. Covert criticism includes indirect criticism, for example, statements about the family member´s characteristics, behavior or opinion that could be perceived as disturbing.

Emotional overinvolvement is defined as a dramatic, exaggerated, or overprotective attitude toward the patient [13] and refers to the self-sacrificing and overprotective attitudes of mothers. Participants were rated as having high expressed emotion if any of the following criteria were met even once: emotional overinvolvement (self-sacrifice/overprotection or nonverbal signs of overinvolvement, e.g., crying), criticism (overall negative relationship or one critical statement/two covert critical statements), or both criticism and emotional overinvolvement [28, 31, 32]. Participants were rated as having low expressed emotion if there was no occurrence of criticism and emotional overinvolvement. Thus, participants could differ in why they were rated as high expressed emotion. For instance, person A shows criticism only once, and person B meets no criteria of criticism but continuously shows all criteria of overinvolvement such as constantly showing emotional display and making more than five positive remarks, both Person A and B are rated as high expressed emotion. (for a more detailed description see Magaña and colleagues [28]. In previous studies and samples, the FMSS proved to have proper predictive validity [33] and Sher-Censor [34] concluded from their review on child FMSS studies good psychometric characteristics of the procedure and coding [34].

Interrater reliability was calculated with 70 samples; the kappa coefficient for expressed emotion level (high vs. low) was very good (κ = 0.93, p < 0.01). Disagreements between raters were solved through discussion until consensus was reached.

#### Procedure

Participants were recruited by collaborating with child and adolescent in- and outpatient psychiatric and psychotherapy clinics and schools in Germany. Inclusion criteria were an age between 12 and 20 years and female gender, as NSSI is mainly associated with the female gender [35]. The inpatient clinics were instructed to inform the participants at admission about the study and ask for their consent to participate. Nonclinical control adolescents were recruited from different high schools. The age group of adolescents recruited was between twelve and 20 years. Before we visited the schools, teachers were given detailed information about the study and informed consent forms to be signed by the parents if the students participated. After obtaining written informed consent from the adolescents and caregivers, clinical interviews were performed in the in- and outpatient clinics for the NSSI and CC samples and in schools for the NC group. Self-report questionnaires were completed on-site or at home and returned by mail. All participants, that is, adolescents and mothers, were informed about the study and gave their written informed consent in accordance with the Declaration of Helsinki. The local ethics committee approved the study.

### Data analyses

We carried out chi-square analyses to assess whether there were differences in expressed emotion across the three samples (NSSI, CC, and NC). To investigate group differences (NSSI vs CC vs NC) in the number of comments regarding FMSS dimensions of expressed emotion, we used a multivariate analysis of variance (MANOVA). Post hoc tests were conducted to analyze pairwise comparisons. The conservative Bonferroni correction was used to control for multiple comparisons, aiming to compensate for possible limitations due to the small sample size. Effect sizes (Cohen’s d) were calculated to further analyze significant group differences. Pearson product-moment correlation coefficients were used to explore associations between adolescent and maternal high expressed emotion status. All analyses were performed using SPSS version 25. Significance levels were set at α = 0.05.

## Results

### Adolescent expressed emotion toward mothers

Compared to the NC group, significantly more participants in the NSSI (61.9% vs. 6.3%, p < 0.01, Cramer’s V = 0.60) and CC (41.2% vs. 6.3%, p < 0.01, Cramer’s V = 0.42) groups met criteria for high expressed emotion (Table 1). The observed differences remained significant using the Bonferroni corrections (α/5 = 0.01). Adolescents with NSSI and CC adolescents did not differ regarding expressed emotion status (61.9% vs. 41.2%, p = 0.20). Across all groups, adolescents with high expressed emotions were predominately related to criticism expressed toward mothers. Only two adolescents in the NSSI group reported emotional overinvolvement. A multivariate analysis comparing the number of comments for expressed emotion dimensions revealed significant main effects of the group for covert criticism, critical tone, and positive relationship (see Table 2). Post hoc analyses showed that adolescents with NSSI exhibited significantly more covert criticism than CC (p < 0.05, d = 0.65) and NC (p < 0.01, d = 1.30) adolescents, while CC and NC adolescents did not significantly differ. The same pattern emerged for critical tone, with adolescents in the NSSI group reaching higher scores than CC (p < 0.01, d = 1.10) and NC (p < 0.01, d = 1.10) adolescents. Comments describing a positive mother-daughter relationship were more common among NC adolescents than adolescents in the NSSI (p < 0.01, d = 1.49) and CC (p < 0.05, d = 0.82) groups.

**Table 1:** Adolescent and maternal expressed emotion levels in the three participant groups (NSSI, CC, NC) assessed with the five-minute speech sample (FMSS)

Variable	Adolescents						χ^2^	*df*	Cramer’s V	Mothers					
	NSSI (*n* = 21)		CC (*n* = 17)		NC (*n* = 32)					NSSI (*n* = 10)		CC (*n* = 7)		NC (*n* = 7)	
	*n*	%	*n*	%	*n*	%				*n*	%	*n*	%	*n*	%
EE status							18.03**	2	0.52						
Low EE	8	38.1	10	58.8	28	87.5				5	50	5	71.4	7	100
High EE	13	61.9	7	41.2	2	6.3				5	50	2	28.6	0	0
HEE subtype							1.52	4	0.19						
Criticism	11	84.6	7	100	2	100				4	80	2	100	0	0
EOI	1	7.7	0	0	0	0				0	0	0	0	0	0
Criticism & EOI	1	7.7	0	0	0	0				1	20	0	0	0	0

*CC* clinical control; *EE* expressed emotion; *HEE* high expressed emotion; *EOI* emotional overinvolvement; *NC* nonclinical control; *NSSI* adolescents who engage in nonsuicidal self-injury

^**^p < 0.01

**Table 2:** Mean number of adolescents’ and mothers’ comments in the three participant groups (NSSI, CC, NC) in the five-minute speech sample (FMSS)

Comment	Adolescents						*F*(2, 65)	η^2^	Mothers					
	NSSI (*n* = 21)		CC (*n* = 17)		NC (*n* = 32)				NSSI (*n* = 10)		CC (*n* = 7)		NC (*n* = 7)	
	*M*	*SD*	*M*	*SD*	*M*	*SD*			*M*	*SD*	*M*	*SD*	*M*	*SD*
Covert criticism	2.00	2.15	0.88	1.11	0.23	0.43	10.72**	0.25	1.10	0.99	0.86	0.90	0.29	0.49
Critical tone	1.24	1.55	0	0	0.13	0.43	11.89**	0.27	1.30	1.25	0.71	1.50	0.86	1.46
Positive relationship	1.76	2.07	3.65	2.34	6.20	3.53	15.20**	0.32	0.50	0.85	2.43	2.07	1.29	1.38

*CC* clinical control; *EE* expressed emotion; *EOI* emotional overinvolvement; *NC* nonclinical control; *NSSI* adolescents who engage in nonsuicidal self-injury

^**^p < 0.01

### Maternal expressed emotion

In the NSSI group, 50% of mothers fulfilled the criteria for high expressed emotion, followed by 28.6% in the CC group. None of the mothers in the NC group was rated as having high expressed emotion. Across all mothers, emotional overinvolvement was rated only once and in combination with criticism in the NSSI group.

### Concordance between adolescent and maternal expressed emotion status

Moderate concordance was found between daughters’ and mothers’ expressed emotion status, with a moderate agreement (Cohen’s κ = 0.42) in n = 24 mother-daughter dyads.

For high expressed emotion, we found a slight agreement for mother-daughters concordance (Cohen’s κ = 0.02, n = 10) for the NSSI group, a moderate agreement for the clinical control group (Cohen’s κ = 0.46, n = 7), and no agreement for the nonclinical control group (Cohen’s κ = 0.0, n = 7). Regarding concordances for low expressed emotions, we found equivalent kappa values with Cohen’s κ = 0.02 (n = 10) for the NSSI group, Cohen’s κ = 0.46 (n = 7) for the clinical control group, and Cohen’s κ = 0.0 (n = 7) for the nonclinical control group.

## Discussion

The present study investigated expressed emotion among adolescents with NSSI, and CC and NC adolescents. To date, this is the first study studying both maternal expressed emotion toward adolescents with NSSI, CC, and NC, as well as adolescents’ expressed emotion toward their mothers.

We found that adolescents’ high expressed emotion was more common in the NSSI and CC groups than in the NC group, supporting the findings that high expressed emotion is associated with psychopathology [20, 27]. Adolescents with NSSI expressed significantly more covert criticism and critical tone toward their mothers than CC and NC adolescents. This is in line with previous studies suggesting that mother-daughter interactions of adolescents with NSSI are characterized by anger and conflict [9]. Crowell et al. [9] reported that impulsive and emotionally sensitive adolescents may experience difficulty inhibiting extreme emotions when faced with high expressed emotions by family members. The greater difficulties with impulse control of adolescents with NSSI compared to CC adolescents [36] may explain the higher level of covert criticism and critical tone toward mothers among adolescents with NSSI. Further, adolescents with NSSI reported less emotional clarity than CC adolescents, underlining the positive relationship between difficulty in identifying emotions and NSSI [37].

As expected, the concordance between adolescent and maternal expressed emotion status in the present study was moderate. This is in line with several studies indicating that informants' reports correlate at low-to-moderate levels [38, 39]. Nevertheless, the significant correlation between adolescent and maternal high expressed emotion in the present study suggests a reciprocal relationship between maternal and adolescent expressed emotion in NSSI. Expressed emotion research on bulimia nervosa indicated that the match in parent and adolescent expressed emotion status may impact treatment outcome. The smallest symptom reduction was reported for the group in which patients showed high expressed emotion and parents low expressed emotion [26]. Therefore, future studies should analyze different family profiles (high expressed emotion mother/high expressed emotion adolescent, high expressed emotion mother/low expressed emotion adolescent, high expressed emotion adolescent/low expressed emotion mother, and low expressed emotion mother/low expressed emotion adolescent) and their impact on the course of NSSI and treatment outcome. Longitudinal studies are needed to understand the direction of the association between maternal and adolescent high expressed emotion and NSSI.

According to the comprehensive model of NSSI [4, 40], maternal high expressed emotion reflects a distal risk factor for NSSI, while high expressed emotion exhibited by adolescents can be understood as a result of adolescent emotion regulation difficulties and poor communication and problem-solving skills. However, a child’s psychopathological symptoms may also elicit expressed emotion from their parents. In addition, results from a longitudinal study examining perceived expressed emotion showed that both internalizing and externalizing symptoms in adolescents predicted adolescents’ perception of maternal expressed emotion, as well as mothers’ self-rated criticism over time [41]. Since NSSI is associated with various mental disorders [42], there might be differences when including mental disorders as moderators between expressed emotion and NSSI. Future studies should include psychopathology measures associated with NSSI in the analysis. In accordance with previous research [16, 17], adolescent and maternal high expressed emotion status in the present study referred primarily to criticism and only to a small extent to emotional overinvolvement. Self-report data showed that adolescents with NSSI indicated higher parental intrusiveness than adolescents without NSSI [23]. As suggested by Reinecke [17], current measures of emotional overinvolvement may assess different components of emotional overinvolvement.

### Limitations and strengths

Given the exploratory analysis with small sample size and low statistical power (10%) and the uneven distribution of groups, especially in the sample of mothers, the results of the present study must be interpreted with caution. The results are further limited by the fact that expressed emotion was only assessed in female adolescents and mothers and not in male adolescents and fathers. Future studies should include all genders since previous research has found gender differences in the association between maternal criticism and NSSI [11, 22]. Future studies can also explore if adolescents with NSSI are generally more critical (not only more critical to their mothers), considering their high levels of negative affectivity and self-derogation.

The use of a cross-sectional design further limits the conclusions about the direction of the effects between adolescent symptomatology and adolescent/maternal high expressed emotion. We included participants between the ages of 18 and 20 years. Although all adolescents lived in their mother’s home, the mother-daughter relationship of female older adolescents may be different from that of younger adolescents, for example, in aspects related to their self-reliance and independence. The strengths of this study include the use of the FMSS as a reliable and valid measure for expressed emotion, including adolescent and maternal expressed emotion levels and the inclusion of a clinical and non-clinical control group. The definition of emotional overinvolvement in the FMSS, also used in the present study, differs from the definition in the Levels of Expressed Emotion Scale used by Ammerman and Brown (2018). Emotional overinvolvement in the FMSS is defined by the self-sacrificing and overprotective attitudes of mothers as well as nonverbal signs of overinvolvement during the monologue (e.g., crying), while the intrusiveness subscale of the Levels of Expressed Emotion Scale primarily refers to controlling parenting behaviors and intrusions of privacy. Furthermore, perceived parental emotional overinvolvement may not correspond with interviewer-rated emotional overinvolvement; this should be addressed in further studies. Therefore, it remains to be clarified if emotional overinvolvement is influential in the engagement and maintenance of NSSI. Further research is needed to examine the validity of the FMSS and especially the operationalization of emotional overinvolvement in samples of adolescents with expressed emotion.

### Practical implications

Reciprocal processes between adolescents and parents (e.g., high expressed emotion) as well as parental (e.g., heightened stress) and child (e.g., impulsivity) contributors leading to insufficient parent–child interactions should be considered in the maintenance of NSSI. The burden of caring for an adolescent who engages in NSSI [43, 44] and changes in parenting behavior as reactions to the engagement in NSSI, for example, more controlling behavior and rule setting [8, 45] may affect parent–child interactions.

NSSI can be conceptualized as a high-cost communication behavior when other behaviors have failed to elicit a response from the family environment [40]. Considering the three ways in which caregivers can affect child adjustment [46], high levels of expressed emotion represent an interaction style that negatively reinforces emotional arousal. Therefore, the findings suggest that it is important to examine adolescent expressed emotion in addition to parental expressed emotion, to gain a better understanding of the reciprocal parent–child interactions in families of adolescents who engage in NSSI. Effective treatments for NSSI, such as the Dialectical Behavior Therapy for Adolescents (DBT-A) 43–45 or the Emotion Regulation Individual Therapy for Adolescents (ERITA) [47, 48], target emotion regulation and interpersonal functioning, especially within the family, and offer the possibility of including the family or parents in the adolescent’s treatment. Breaking a vicious cycle of interactions between adolescents and parents characterized by frustration and criticism and reestablishing more functional family interactional patterns can reduce high expressed emotion [49]. Given that adolescents who engage in NSSI reported fewer positive aspects in the mother–child relationship than NC adolescents, interventions should not focus merely on reducing the negative relational aspects but also on enhancing the positive aspects of the relationship.

### Summary

This exploratory study aimed to examine levels of expressed emotion in adolescents who engage in NSSI toward their mothers as well as the level of expressed emotion in mothers toward their child who engage in NSSI compared to mother-daughter dyads in clinical and nonclinical control groups. To assess expressed emotion the Five-Minute Speech Sample (FMSS) was used. The sample consisted of 70 female adolescents aged 12–20 years (M = 15.28 years, SD = 1.81; NSSI: n = 21, CC: n = 17, NC: n = 32) and 24 mothers aged 38–56 years (M = 46.47 years, SD = 4.61) who completed the FMSS. In the FMSS, daughters and mothers were asked to speak for five uninterrupted minutes about their mother/daughter and how they get along with each other. Adolescents who engaged in NSSI exhibited significantly more covert criticism and critical tone toward their mothers than adolescents in the clinical control and non-clinical control groups. The findings highlight the importance of family-based interventions in the treatment of NSSI to enhance a positive relationship between parents and adolescents.

## Data Availability

The datasets used and/or analyses during the current study are available from the corresponding author on reasonable request.
